# Appendectomy and risk of Parkinson’s disease: a systematic review and meta-analysis

**DOI:** 10.3389/fneur.2025.1619236

**Published:** 2025-07-21

**Authors:** Hok Leong Chin, Yiu Sing Tsang, Haojun Shi

**Affiliations:** 1 Section of Neurology, Department of Pediatrics, University of Chicago Medicine, Chicago, IL, United States; 2 Department of General Surgery, Ruijin Hospital, Shanghai Jiao Tong University, Shanghai, China; 3 Department of Pancreatic Surgery, Pancreatic Disease Institute, Huashan Hospital, Fudan University, Shanghai, China

**Keywords:** Parkinson’s disease, Parkinson, appendectomy, systematic review, meta-analysis

## Abstract

**Introduction:**

Braak’s hypothesis suggests that α-synuclein may enter the central nervous system through the enteric nervous system and contribute to the pathogenesis of Parkinson’s disease (PD). The appendix, enriched in α-synuclein, has been proposed as a possible entry point in PD pathogenesis. This systematic review and meta-analysis aimed to assess the association between appendectomy and PD risk using newly available data.

**Methods:**

A literature search was conducted in PubMed and Embase through September 10, 2024, to identify studies on appendectomy and PD risk. Two independent reviewers screened and assessed articles for eligibility with a third reviewer involved in cases of disagreement. Study quality was assessed using the Newcastle-Ottawa Scale. Data for meta-analysis were pooled using a random-effects model and analyzed in Review Manager 5.4. Meta-regression, subgroup, and sensitivity analyses were performed.

**Results:**

Nine studies met inclusion criteria. Meta-analysis indicated no significant association between appendectomy and PD risk (RR: 1.01, 95% CI: 0.90–1.12, *p* = 0.89). Subgroup analyses showed similar findings. Sensitivity analyses did not change the estimate.

**Conclusion:**

This analysis suggests no association between appendectomy and PD risk.

## 1 Introduction

Parkinson’s disease (PD) is the second most common neurodegenerative disorder globally, after Alzheimer’s disease (1). Clinically, PD is characterized by bradykinesia, tremor, rigidity, and postural instability, along with various non-motor symptoms (2). The Braak’s hypothesis was previously proposed, suggesting α-synuclein may enter the brain through the olfactory and enteric nervous system, potentially leading to sporadic PD (3–5). The appendix is notably enriched in α-synuclein compared to other gastrointestinal structures, potentially serving as an anatomical entry point in PD pathogenesis (6). Therefore, appendectomy can potentially impact the pathogenic development of PD. Previous observational studies investigating the association between appendectomy and PD risk have yielded inconsistent results (7, 8). This study aimed to reassess this association in light of newly available literature.

## 2 Methods

This study was conducted in accordance with the Preferred Reporting Items for Systematic Reviews and Meta-Analyses (PRISMA) guidelines (9). The study was registered on INPLASY per protocol to promote transparency and reduce potential bias (Registration number: INPLASY202490039).

### 2.1 Literature search and inclusion criteria

A comprehensive literature search was conducted in the electronic databases PubMed and Embase through September 10, 2024, to identify potential literature. The search terms used were (parkinson OR parkinsonian OR parkinsonism OR parkinson disease OR parkinson’s disease OR paralysis agitans OR parkinsonian disorders OR parkinsonian syndromes OR parkinsonian diseases) AND (appendectomy OR appendectomy OR appendicitis OR appendix OR append*). Inclusion criteria encompassed case–control studies, prospective cohort studies, and retrospective cohort studies published in English, of high quality, with a matched control group, and reporting measurable outcomes.

### 2.2 Data extraction

HubMeta, a free web-based data entry system, was used in the data extraction process. Two independent reviewers (HLC and YST) screened titles and abstracts of extracted data after removing duplicates. Full-text articles were then assessed independently by the same reviewers to determine eligibility. Disagreements were resolved through discussion with a third reviewer (HS) until consensus was reached.

### 2.3 Quality assessment

The quality of the collected literature was assessed using the Newcastle-Ottawa Scale (NOS). Studies with a score > = 7 were considered high quality studies. Two researchers (HLC and YST) independently conducted the quality assessments, with any disagreements resolved by a third reviewer (HS) after discussion.

### 2.4 Statistical analysis

We pooled the data and calculated adjusted relative risks (RR) with 95% Confidence Interval (95% CI). Odd ratios (OR) and Hazard ratios (HR) were treated as RR in this study, given that the prevalence of PD in the general population is less than 10% (10). The meta-analysis study employed the random-effects model, and statistical analyses were conducted using Review Manager 5.4 (Nordic Cochrane Centre, Copenhagen, Denmark). A *p*-value <0.05 was considered statistically significant. Heterogeneity was evaluated using the *I*^2^ statistic, with *I*^2^ > =50 indicating significant heterogeneity. Subgroup analyses were conducted using a fixed-effects model to assess differences between groups. Initial subgroup analyses included maximum follow-up years and study design. Additional subgroup analyses based on geographic region and appendectomy assessment method were conducted in response to reviewers’ feedback. No adjustment for multiple testing was applied for subgroup analyses. Sensitivity analysis was also performed to determine the robustness of the results. Meta-regression, Egger’s test and Begg’s test were conducted using STATA/SE version 17.0 (StataCorp, College Station, TX, USA). Meta-regression was performed as a random-effects meta-regression model with restricted maximum likelihood (REML) method. The moderators included follow-up years, study design, geographic region, and appendectomy assessment method.

## 3 Results

### 3.1 Study selection and characteristics

**Figure 1:**
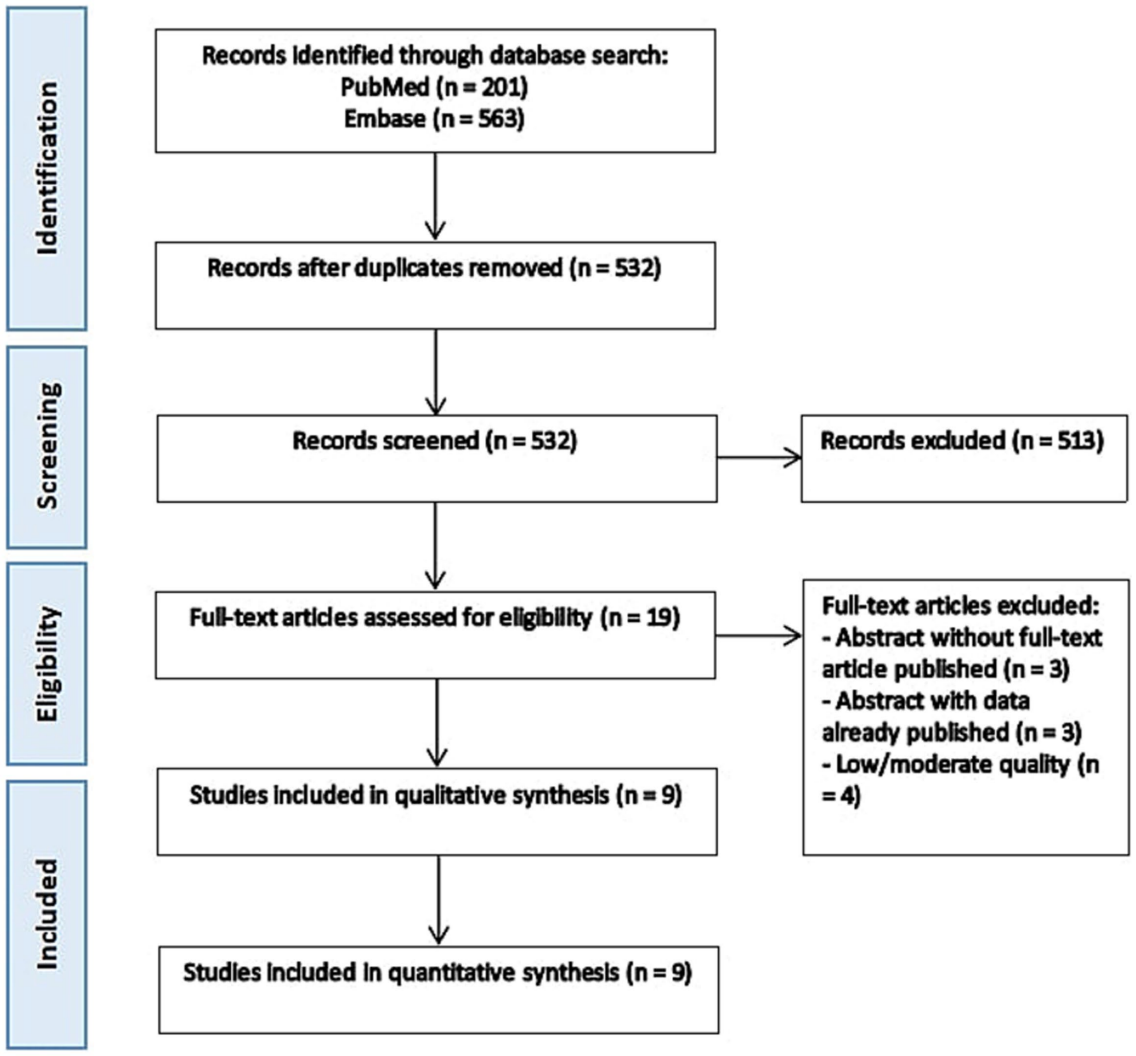
Flow diagram of included studies.

The initial literature search retrieved 764 articles, with 532 remaining after removing duplicates. Title and abstract screening excluded 513 articles, and 19 full-text articles were assessed for eligibility. Of these, three articles had only abstract available without further data published in full text. Three articles were abstracts that later published as full articles which were included in the analysis. Four studies were excluded based on quality criteria assessed by NOS. Ultimately, 9 studies met the inclusion criteria for the systematic review and meta-analysis (11–19) (Figure 1). The quality assessment of the included studies using the Newcastle-Ottawa Scale is depicted in Table 1.

**Table 1:** Quality assessment of the included studies by the Newcastle-Ottawa Scale.

Articles	Selection				Comparability	Outcome			Total
	Representativeness of exposed cohort	Selection of nonexposed cohort	Ascertainment of exposure	Outcome not present at the start of the study		Assessment of outcomes	Length of follow-up	Adequacy of follow-up	
Marras et al. (11)	*	*	*	*	**	*	*		8
Svensson et al. (12)	*	*	*	*	**	*	*		8
Killinger et al. (13)	*	*	*	*	**	*	*		8
Palacios et al. (14)		*		*	**	*	*	*	7
Liu et al.^#^ (15)	*	*	*	*	**	*	*		8
Jain et al. (16)	*	*	*	*	**	*	*	*	9
Koning et al. ^#^ (17)	*	*	*	*	**	*	*		8
Park et al. (18)	*	*	*	*	**	*	*		8
Wang et al. (19)	*	*		*	**		*	*	7

NOS In case–control studies consists of: Selection: (1) Representativeness of the cases, (2) Case definition adequacy, (3) Selection of controls, (4) Definition of controls; Comparability; Exposure: (1) Ascertainment of exposure, (2) Same method of ascertainment for cases and controls, (3) Non-response rate.

The included studies comprised a total population of 8,297,621, with sample sizes ranging from 49,248 to 3,224,650. The studies were published between 2016 and 2024 and included participants from Canada (11), Denmark (12), Sweden (13, 15), United States (14, 16, 17), Korea (18), United Kingdom (19). Of the 9 included studies, 7 studies were cohort studies (11–14, 16, 18, 19), 1 was case–control (15), and 1 employed a case–control design with complementary cohort (17). Assessment of appendectomy included self-report and recorded codes. Assessment of PD included recorded codes and history of antiparkinson drug prescription. Maximum follow-up time ranged from 13 years to 52 years. All included studies scored highly on the NOS, with scores between 7 and 9. The characteristics of included studies are depicted in Table 2.

**Table 2:** Details of included studies.

Articles	Country	Data information	Study design	Sample size	Appendectomy assessment	PD assessment	Maximum follow-up years	Effect estimate (95% CI)	Adjustments	Study quality
Marras et al. (11)	Canada	Canadian Institute for Health Information (CIHI) database and Ontario Health Insurance Plan (OHIP) database	Cohort	85,994	Medical record	ICD-8,9,10 codes and antiparkinson drug prescription	17	HR 1.004 (0.740–1.364)	Median neighborhood income and Aggregated Diagnosis Groups	8
Svensson et al. (12)	Denmark	Danish National Patient Registry (DNPR)	Cohort	1,594,548	Operation codes	Record from DNPR using ICD-8,10 codes	34	HR 1.14 (1.03–1.27)	Age, sex, smoking, head trauma, diabetes, cardiovascular diseases, Charlson Comorbidity Index, ulcerative colitis, and Crohn’s disease	8
Killinger et al. (13)	Sweden	Swedish National Patient Registry (SNPR) and Parkinson’s Progression Markers Initiative (PPMI)	Cohort	1,698,000	ICD codes	ICD-7,8,9,10 codes	52	OR 0.831 (0.756–0.907)	Sex and urban/rural municipality	8
Palacios et al. (14)	United States	Nurses’ Health Study (NHS) and Health Professionals Follow-up Study (HPFS)	Cohort	138,698	Self-report	Medical record	26	HR 1.08 (0.94–1.23)	Age, smoking, and pack-years smoking. Additional adjustment for postmenopausal hormone use in NHS	7
Liu et al. (15)	Sweden	Swedish National Patient Registry (SNPR) and Swedish Population and Housing Censuses	Case–control	3,224,650	ICD codes	ICD-7,8,9,10 codes	46	OR 0.84 (0.80–0.88)	Birth year, sex, country of birth, highest achieved education, chronic obstructive pulomonary disease, comorbidity index, and number of hospital visits	8
Jain et al. (16)	United States	Medicare data	Cohort	329,976	ICD codes	ICD-9,10 codes	15	HR 0.916 (0.861–0.976)	Age, race, sex, comorbidities, cancers, socio-economic status, provider visits, count of visits, and residents of States	9
Koning et al. (17)	United States	TriNetX medical record	Combined case–control and cohort	49,248	TriNetX codes	ICD-10 code with documented ambulatory visit and antiparkinson drug prescription	16	OR 2.40 (1.15–5.02)	Prodromal motor and non-motor PD symptoms and Charlson Comorbidity index	8
Park et al. (18)	Korea	National Health Insurance Service-National Sample Cohort (NHIS-NSC)	Cohort	703,831	Procedure codes	ICD-10 code and registration code for government co-payment	13	HR 1.42 (0.88–2.30)	Age, sex, diabetes mellitus, hypertension, and smoking	8
Wang et al. (19)	United Kingdom	UK Biobank	Cohort	472,676	Not reported, obtained from UK Biobank	Not reported, obtained from UK Biobank	16	HR 1.120 (1.016–1.234)	Age, gender, ethnicity, education level, alcohol intake, smoking, body mass index, Townsend deprivation index, hypertension, and Polygenic Risk Score	7

### 3.2 Meta-analysis for appendectomy and risk of PD

**Figure 2:**
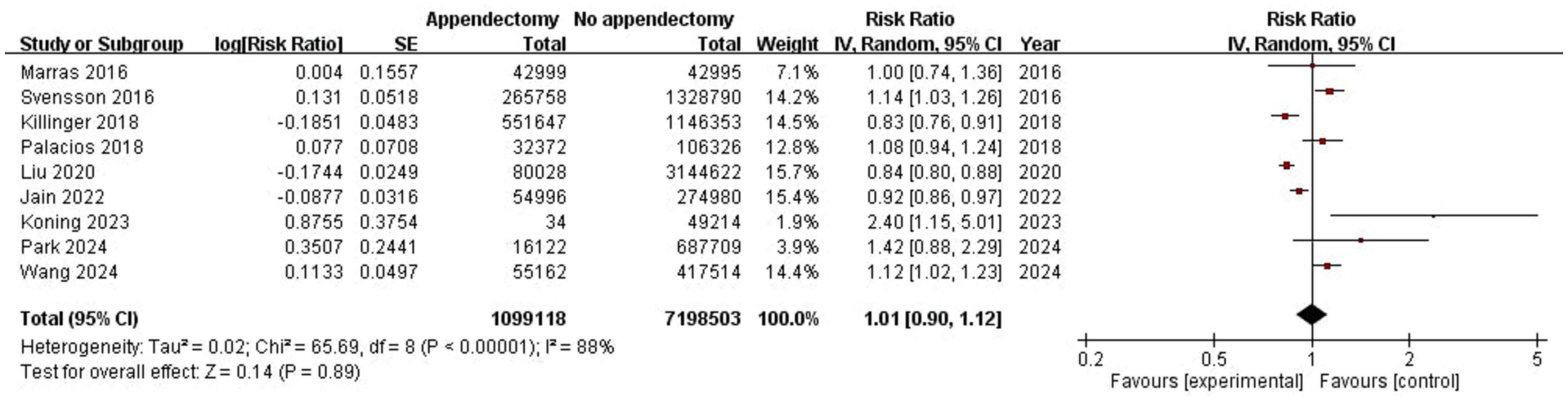
Meta-analysis forest plot of appendectomy and risk of PD.

**Figure 3:**
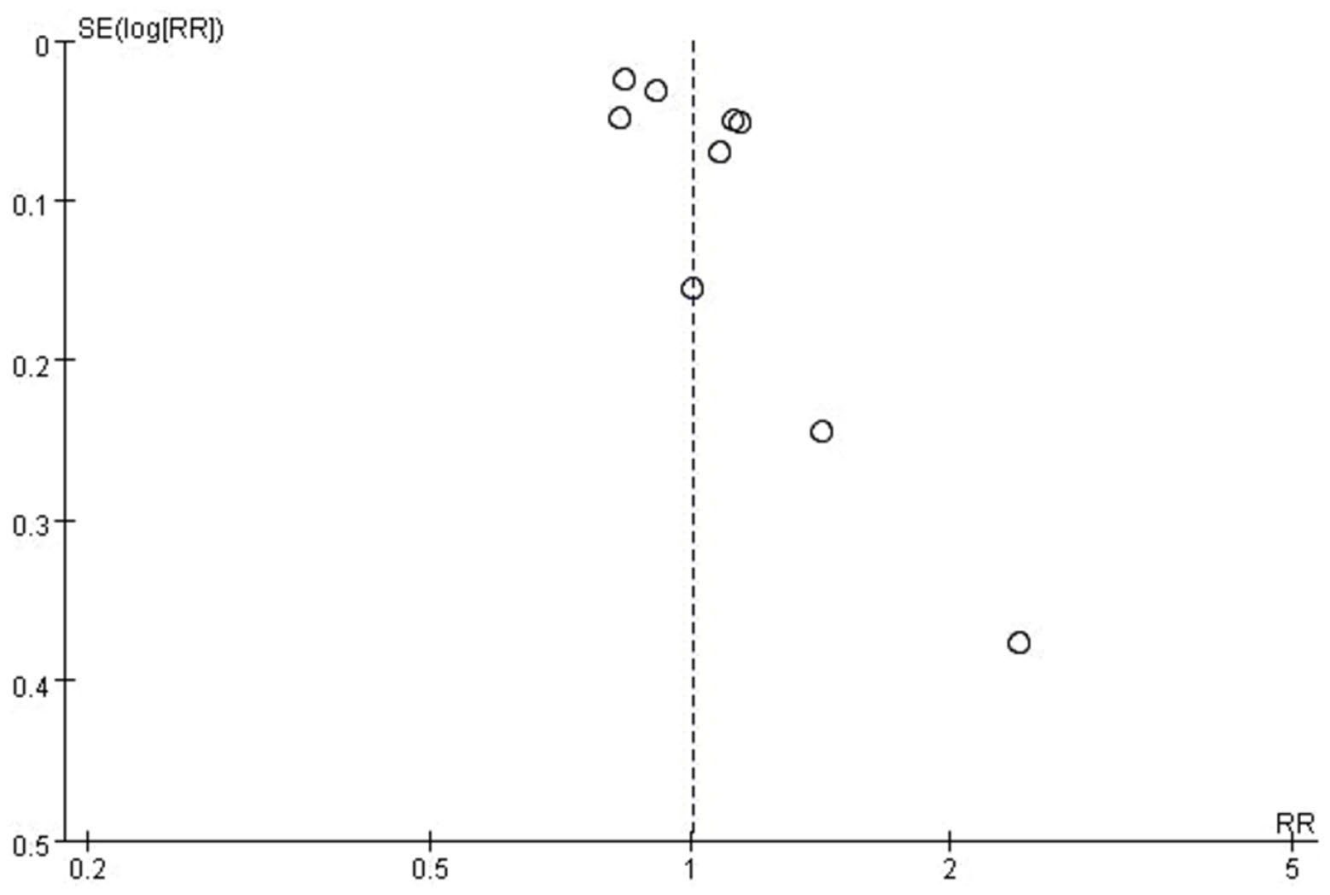
Funnel plot of the meta-analysis of the included studies.

Pooled results from the 9 included studies demonstrated no statistically significant association between appendectomy and risk of PD (Pooled RR: 1.01, 95%CI: 0.90–1.12, *p* = 0.89) (Figure 2). Significant heterogeneity was observed (*I*^2^ = 88%, *p* < 0.01). The funnel plot appeared asymmetrical, supported by a positive Egger’s test (*p* < 0.01), while Begg’s test was not significant (*p* = 0.18), suggesting the presence of potential small-study effects (Figure 3). Meta-regression analyses were conducted to evaluate potential moderators, including follow-up years, study design, geographic region, and appendectomy assessment method; none of these variables sufficiently explained the heterogeneity observed (all *p* > 0.05).

### 3.3 Subgroup analyses of appendectomy and risk of PD

Given that PD is a chronic disease that becomes more common with age, and all effect estimates were treated as RR due to PD prevalence being <10% in the general population, two subgroup analyses were decided to be performed before the beginning of the study (Table 3).

**Table 3:** Subgroup analyses for appendectomy and risk of PD.

Subgroup	Number of studies	RR (95% Cl)	*I^2^* static	Heterogeneity *p*	Overall effect *p*	Subgroup differences *p*
Maximum follow-up years						
>30	3	0.88 (0.84–0.91)	93%	<0.01	<0.01	<0.01
<=30	6	0.99 (0.95–1.04)	77%	<0.01	0.73	
Study design						
Cohort	7	0.98 (0.94–1.02)	83%	<0.01	0.30	<0.01
Case–control	2	0.84 (0.80–0.89)	87%	<0.01	<0.01	
Geographic region						
Asia-Pacific	4	0.93 (0.88–0.99)	70%	0.02	0.02	0.73
Europe	5	0.92 (0.89–0.95)	93%	<0.01	<0.01	
Appendectomy assessment method						
ICD codes	5	0.91 (0.88–0.94)	92%	<0.01	<0.01	0.03
Non ICD	4	1.12 (0.93–1.36)	50%	0.11	0.23	

For maximum follow-up years, studies were divided into two subgroups: >30 years and <30 years. A statistically significant subgroup differences *p*-value was observed, suggesting a possible presence of subgroup effect. However, substantial amount of heterogeneity was noted within both subgroups (>30 years: *I*^2^ = 93%, *p* < 0.01; <=30 years: *I*^2^ = 77%, *p* < 0.01), making the validity of effect estimate for each subgroup uncertain (Table 3).

For study design, studies were divided into two subgroups: cohort and case–control. One of the included studies used a design of case–control with complementary cohort. This study was treated as a case–control design in our study. A statistically significant subgroup differences *p*-value was observed, suggesting a possible presence of subgroup effect. However, substantial amount of heterogeneity was noted within both subgroups (cohort: *I*^2^ = 83%, *p* < 0.01; case–control: *I*^2^ = 87%, *p* < 0.01), making the validity of effect estimate for each subgroup uncertain (Table 3).

Additional subgroup analyses based on geographic region and appendectomy assessment method were conducted in response to reviewers’ feedback, which also demonstrated high heterogeneity within geographic region subgroups (Asia-Pacific: *I*^2^ = 70%, *p* = 0.02; Europe: *I*^2^ = 93%, *p* < 0.01) as well as within appendectomy assessment method subgroups (ICD codes: *I*^2^ = 92%, *p* < 0.01; Non ICD codes: *I*^2^ = 50%, *p* = 0.11). A statistically significant subgroup difference was observed in the appendectomy assessment method analysis (Table 3).

### 3.4 Sensitivity analyses of appendectomy and risk of PD

Sensitivity analyses were performed to evaluate the robustness of findings to changes. Each study was omitted one by one in performing the sensitivity analyses. Since the two Swedish studies included had a potential partial overlap of populations with variations in ascertainment, a model excluding both studies was also performed to assess the potential effect of oversampling on skewing the results (13, 15). Results indicated that removing any single study did not significantly alter the conclusion that no association was observed between appendectomy and the risk of PD (Table 4).

**Table 4:** Sensitivity analyses for appendectomy and risk of PD.

Study omitted	RR (95% Cl)	*I^2^* static	Heterogeneity *p*	Overall effect *p*
Marras et al. (11)	1.01 (0.90–1.13)	89%	<0.01	0.88
Svensson et al. (12)	0.98 (0.88–1.09)	85%	<0.01	0.73
Killinger et al. (13)	1.04 (0.92–1.18)	88%	<0.01	0.49
Palacios et al. (14)	1.00 (0.89–1.12)	88%	<0.01	0.97
Liu et al. (15)	1.04 (0.93–1.17)	83%	<0.01	0.49
Jain et al. (16)	1.04 (0.90–1.19)	89%	<0.01	0.60
Koning et al. (17)	0.99 (0.89–1.10)	88%	<0.01	0.84
Park et al. (18)	0.99 (0.89–1.11)	89%	<0.01	0.90
Wang et al. (19)	0.99 (0.88–1.10)	86%	<0.01	0.79
Killinger et al. and Liu et al. (13, 15)	1.09 (0.96–1.23)	78%	<0.01	0.17

## 4 Discussion

This systematic review and meta-analysis comprehensively evaluated the relationship between appendectomy and the risk of PD, analyzing data from nine observational studies involving a combined population size of approximately 8 million individuals. Our findings suggest no statistically significant association between appendectomy and the risk of PD. These results align with two previously reported meta-analyses on this topic from 2019 and 2020 (7, 8). Compared to previous meta-analyses, our study included additional studies and doubled the number, providing stronger evidence with newly available data (15–19). Notably, our analysis also incorporated one Asian study (18), addressing a gap in previous studies, which focused primarily on European and North American populations. Additionally, we applied a more rigorous quality criterion compared to previous reviews, including only articles with a Newcastle-Ottawa Scale (NOS) score of > = 7.

Braak’s hypothesis proposed that PD may originate in the gut, with synucleinopathy transported retrogradely to the central nervous system, ultimately leading to PD (3). However, this hypothesis remains controversial. Some neuropathological studies have questioned Braak’s hypothesis, as the observed distribution pattern of synucleinopathy does not always align with it, suggesting that it may not sufficiently explain PD pathogenesis (20, 21). Although the appendix mucosa contains abundant α-synuclein, potentially serving as a reservoir for spread to the brain, our study did not support a protective effect of appendectomy against PD. While Braak’s hypothesis encompasses a broader range of proposed entry sites and mechanisms, our epidemiologic findings suggest that the appendix may play a less prominent role as an entry point in the pathogenesis of PD. Given PD’s lengthy prodromal period and the gradual development of pathology in the gastrointestinal tract, subgroup analyses by follow-up years were also performed, revealing consistent results with no observed differences between subgroups (22).

This systematic review and meta-analysis has some limitations. Despite including studies with large populations and high-quality scores (NOS > =7), the study pool was relatively small and primarily focused on Western, developed countries, limiting generalizability and the power of publication bias assessment. Publication bias and substantial methodological variability, such as differences in how appendectomy and PD were defined and assessed, were present across the included studies. Additionally, differences in adjusted confounders across studies limited the comparability among studies. These may contributed to the observed heterogeneity. Despite conducting subgroup analyses and meta-regression, no consistent moderators could be identified. Furthermore, while subgroups analyses offer valuable exploratory insights, these also raised risk of type I error. Results from subgroup analyses should be regarded exploratory and hypotheses generating rather than confirmatory.

## Data Availability

The original contributions presented in the study are included in the article, further inquiries can be directed to the corresponding author.
