# Correction: Appendectomy and risk of Parkinson's disease: a systematic review and meta-analysis

**DOI:** 10.3389/fneur.2026.1773653

**Published:** 2026-01-29

**Authors:** Hok Leong Chin, Yiu Sing Tsang, Haojun Shi

**Affiliations:** 1 Section of Neurology, Department of Pediatrics, University of Chicago Medicine, Chicago, IL, United States; 2 Department of General Surgery, Ruijin Hospital, Shanghai Jiao Tong University, Shanghai, China; 3 Department of Pancreatic Surgery, Pancreatic Disease Institute, Huashan Hospital, Fudan University, Shanghai, China

**Keywords:** Parkinson's disease, Parkinson, appendectomy, systematic review, meta-analysis

In the published article, author Yiu Sing Tsang was erroneously omitted from first authorship and equal contribution designation, which should have been shared with author Hok Leong Chin.

In the affiliations section author Haojun Shi was erroneously only assigned with affiliation 3 “Department of Pancreatic Surgery, Pancreatic Disease Institute, Huashan Hospital, Fudan University, Shanghai, China”. The correct affiliations for author Haojun Shi are affiliation 2 “Department of General Surgery, Ruijin Hospital, Shanghai Jiao Tong University, Shanghai, China and affiliation 3.

The original version of this article has been updated.

